# Evaluation of association tests for rare variants using simulated data sets in the Genetic Analysis Workshop 17 data

**DOI:** 10.1186/1753-6561-5-S9-S86

**Published:** 2011-11-29

**Authors:** Wenan Chen, Xi Gao, Jiexun Wang, Chuanyu Sun, Wen Wan, Degui Zhi, Nianjun Liu, Xiangning Chen, Guimin Gao

**Affiliations:** 1 Department of Biostatistics, Virginia Commonwealth University School of Medicine, 830 East Main Street, One Capitol Square, 7th Floor, Richmond, VA 23298-0032, USA; 2 Department of Computer Science, Virginia Commonwealth University, 401 West Main Street, Room E4225, PO Box 843019, Richmond, VA 23284-3019, USA; 3 Department of Biostatistics, School of Public Health, University of Alabama at Birmingham, 1665 University Boulevard, Birmingham, AL 35294, USA; 4 Departments of Psychiatry, Virginia Commonwealth University School of Medicine, Richmond, VA 23298-0003, USA

## Abstract

We evaluate four association tests for rare variants—the combined multivariate and collapsing (CMC) method, two weighted-sum methods, and a variable threshold method—by applying them to the simulated data sets of unrelated individuals in the Genetic Analysis Workshop 17 (GAW17) data. The family-wise error rate (FWER) and average power are used as criteria for evaluation. Our results show that when all nonsynonymous SNPs (rare variants and common variants) in a gene are jointly analyzed, the CMC method fails to control the FWER; when only rare variants (single-nucleotide polymorphisms with minor allele frequency less than 0.05) are analyzed, all four methods can control FWER well. All four methods have comparable power, which is low for the analysis of the GAW17 data sets. Three of the methods (not including the CMC method) involve estimation of *p*-values using permutation procedures that either can be computationally intensive or generate inflated FWERs. We adapt a fast permutation procedure into these three methods. The results show that using the fast permutation procedure can produce FWERs and average powers close to the values obtained from the standard permutation procedure on the GAW17 data sets. The standard permutation procedure is computationally intensive.

## Background

Genome-wide association studies have become a useful tool for identifying the genetic variants that influence complex diseases or traits [1]. Traditional association tests can perform effectively for the common variant/common disease model [2], but they have low power to detect rare variants. The major reasons are that rare variants have very low minor allele frequencies (MAFs) (e.g., less than 1% or less than 5%) and have high allelic heterogeneity. Studies have shown that rare variants can contribute significantly to common diseases [3], and several methods designed for rare variants have been developed [4-6]. Li and Leal [4] proposed a combined multivariate and collapsing (CMC) method that groups and collapses rare variants. Madsen and Browning [5] proposed two weighted-sum methods that combine rare variants into a functional unit by using a weighted approach. Price et al. [6] extended these methods by using a variable threshold method that selects an optimal threshold and assumes that variants with MAFs below this threshold are substantially more likely to be functional. These four methods are reviewed in more details by Dering et al. [7]. The developers of these methods have evaluated their methods using simulation studies. However, the designs of these simulation studies are different from each other. The Genetic Analysis Workshop 17 (GAW17) data provide an opportunity for us to further evaluate these methods in a relatively unbiased manner. In this paper, we compare these four methods using the simulated data sets of unrelated individuals in the GAW17 data.

## Methods

### Evaluation criteria

In this study, we analyze only nonsynonymous single-nucleotide polymorphisms (SNPs) from 22 autosomal chromosomes. The SNPs in each gene are analyzed as a functional unit. For a genome-wide significance level of *α* = 0.05, we use the Bonferroni procedure to adjust the threshold (*α*′) for each gene, *α*′ = *α*/*n*_gene_. Genes with *p-*values less than or equal to *α*′ are treated as significant genes.

### Family-wise error rate

When testing association for a single gene, one can use the type I error rate to measure the false-positive discoveries. For genome-wide gene-based association studies that involve multiple hypothesis testing, we use the family-wise error rate (FWER) to measure the false-positive discoveries. The FWER is the probability of falsely rejecting at least one true null hypothesis. When analyzing the 200 simulated replicates of unrelated individuals in the GAW17 data, we estimate the FWER as FWER = *n_f_* /*n*, where *n_f_* is the number of replicates in which at least one unrelated gene is falsely detected and *n* = 200 is the total number of phenotype replicates of simulated data.

### Average power

For testing a single gene that affects the disease, we can calculate the power of a test. This power is referred to as the per-hypothesis power. For genome-wide gene association studies, we estimate the average power, which is defined as the average of the per-hypothesis powers of tests for the genes that affect the disease [8]. For example, in the GAW17 data with 2,196 genes, if only 10 genes affect the disease status, then the average power is the mean of the per-hypothesis powers of tests for these 10 genes.

### Four association tests for rare variants

We describe briefly the four methods used in this study: the CMC method, the two weighted-sum methods, and the variable threshold method.

The basic idea of the CMC method [4] is to empirically aggregate the rare variants (SNPs) in a functional unit into several subgroups according to their MAFs. Each subgroup is treated as a new single variant in the subsequent multivariate analysis (such as the Hotelling test).

For a case-control data set, weighted-sum method I proposed by Madsen and Browning [5] tests a gene each time. It calculates a genetic score (*γ_j_*) for individual *j* as a weighted sum of the genetic scores of all variants in the gene and then calculates the sum of the ranks for affected individuals as:(1)

where *A* is the population of the affected individuals. For the case-control data set, permuting the affected or unaffected status of the individuals can generate a new data set. Suppose that *k* permuted data sets are generated from the original data set by permutation. Let *x_j_* denote sums of ranks for the *j*th permuted data set (*j* = 1, 2, …, *k*). Let  and  denote the mean and sample standard deviation, respectively, of these *x_j_*. Weighted-sum method I defines a standardized score-sum test statistic:(2)

and assumes that *z* has an approximately standard normal distribution under the null hypothesis. The *p*-value of the test statistic *z* for the observed data is calculated using the normal distribution *N*(0, 1) [5,7].

Weighted-sum method II follows the same steps as weighted-sum method I except that it constructs a test statistic based on the sum of the genetic scores,(3)

in place of the sum of the ranks given by Eq. (1). Madsen and Browning [5] mentioned that these two methods have similar results. Price et al. [6] provides a clear description of weighted-sum method II.

Price et al. [6] proposed a variable threshold method. This method defines a *z*-score statistic and chooses the optimal threshold that maximizes the *z* score. In this method, *p*-values are estimated using a standard permutation procedure.

### Estimating *p*-values using a fast permutation procedure

Except for the CMC method, the other three methods estimate *p*-values using permutation procedures. The standard permutation procedure can be computationally intensive, but the normal approximation procedure described for weighted-sum method I can inflate the type I error rate (see Results section). In this study, we adapt a fast permutation procedure [9] for the three methods. Here, we briefly describe the fast permutation procedure.

In a permutation procedure with *N* permuted data sets, the *p*-value can be estimated using the binomial distribution *B*(*N*, *p*) [10]. Let *X* denote the number of permutations that are as extreme as or more extreme than the observed data. By using Agresti-Coull interval estimation, as recommended by Brown et al. [11], we can estimate the *p*-value as:(4)

with a 100(1 − *β*)% confidence interval [*P_L_*, *P_U_*], where:(5)(6)(7)

and(8)

where  is the inverse of the cumulative distribution function for the standard normal distribution. We set *β* = 0.05 in our study.

In the fast permutation procedure for testing a gene, given the maximum number of permutations *N_m_* (e.g., 10^6^), if for any number 1,000 ≤ *N* ≤ *N_m_* the corresponding lower bound *P_L_* is greater than or equal to *α*′, then the true *p*-value for this gene is unlikely reach the Bonferroni threshold *α*′; therefore we give up the permutation procedure and do not reject the null hypothesis for this gene. This procedure can save computing time significantly. Note that in this paper, for the standard permutation procedure, we use (*X* + 1)/(*N* + 1) to estimate the *p*-value.

### GAW17 data sets

The GAW17 data include 24,487 SNPs from 3,205 genes [12]. We test associations only for the 2,196 genes with nonsynonymous SNPs from the 697 unrelated individuals. In the data there are four phenotypes: Q1, Q2, Q4, and disease status. Q1, Q2, and Q4 are quantitative traits, whereas disease status is a binary trait with the labels affected or unaffected. For each phenotype, there are 200 phenotype replicates with the same underlying genetic information in the simulated data. The three quantitative traits are associated with 9, 15, and 0 genes, respectively. The disease status is related to 36 genes. Because the four methods analyzed in this paper are for case-control designs, in order to use Q1, Q2, and Q4 to evaluate these methods, we generate three new binary traits, *D*_1_, *D*_2_, and *D*_4_ for Q1, Q2, and Q4, respectively. We assign *D_i_* = 1 (disease) for individuals with *Q_i_* (i.e., Q1, Q2, or Q4) among the top 30% percentile and *D_i_* = 0 (control) for the remaining individuals (*i* = 1, 2, 3). The phenotypes *D*_1_, *D*_2_, and disease status are used to calculate the average power, and *D*_4_ is used to calculate the FWER because *D*_4_ is not related to any genes.

## Results

### Evaluation of the fast permutation procedure

To evaluate the performance of the fast permutation procedure, we calculate the FWER and average power of weighted-sum method I, weighted-sum method II, and the variable threshold method when using different permutation procedures to estimate *p-*values based on analyzing the simulated replicates of unrelated individuals with all nonsynonymous SNPs. The permutation procedures include the normal approximation procedure with both 10^3^ and 10^6^ permutations, the fast permutation procedure with the maximum number of permutations *N_m_* = 10^6^, and the standard procedure with 10^6^ permutations. We set the genome-wide significance level to *α* = 0.05. Here we report only the FWER and average power of weighted-sum method I (Table 1). Weighted-sum method II and the variable threshold method have similar results.

From Table 1, we can see that the results from the normal approximation procedures have much higher FWERs than the other procedures. The fast permutation procedure has the lowest FWER: 0.055, which is very close the nominal level 0.05. The computing time of the fast permutation procedure is about one-fourth that of the standard procedure. Table 1 also shows that the power of weighted-sum method I when using the fast permutation procedure is almost the same as the power when using the standard permutation procedure. To some extent, the fast permutation procedure is slightly more conservative than the standard permutation procedure.

**Table 1:** Family-wise error rate (FWER) and average power of weighted-sum method I using different permutation procedures

Permutation procedure^a^	FWER	Power			Computing time (approximate)^b^
		
	*D*_4_	*D*_1_	*D*_2_	Disease	
Normal 10^3^	0.1600	0.1200	0.0123	0.0069	36 minutes
Normal 10^6^	0.1200	0.1200	0.0085	0.0074	16 days
Standard permutation	0.0650	0.1183	0.0065	0.0069	16 days
Fast permutation	0.0550	0.1172	0.0065	0.0065	4.5 days

Data used here are the 200 data sets from GAW17 with all nonsynonymous SNPs.

^a^Normal 10^3^ is the normal approximation with 1,000 permutations; normal 10^6^ is the normal approximation with 10^6^ permutations; standard permutation is the standard permutation procedure with 10^6^ permutations; and fast permutation is the fast permutation procedure with the maximum number of permutations, 10^6^.

^b^All studies are performed in Matlab on a computer with a CPU of 2.66 GHz and 32 GB memory. The computing time is the total running time for all four phenotypes.

Why do the normal approximation procedures in the two weighted-sum methods generate inflated FWERs? One major reason is that the two methods assume that the standardized score-sum test statistics *z* given by Eq. (2) approximately follow the standard normal distribution *N*(0, 1) under the null hypothesis. However, this may not be the case when the two methods are applied to the 200 simulated case-control data sets from the GAW17 data. For these data sets, when we set *k* = 1,000 or even larger, the quantile-quantile plots show that the score-sum test statistics *z* often have distributions that deviate from the normal distribution (data not shown).

### Comparison of the four association methods

Because the fast permutation procedure has good performance, we incorporate it into the two weighted-sum methods and the variable threshold method to estimate *p-*values. To compare the four methods, we apply them to two scenarios: In scenario 1 all nonsynonymous SNPs (rare and common variants) and therefore 2,196 genes are analyzed. Thus *n*_gene_ = 2,196, and the threshold for a single-gene *α*′ = 2.27 × 10^−5^. In scenario 2 only nonsynonymous SNPs with MAF < 0.05 and therefore 1,999 genes are analyzed. The corresponding *n*_gene_ = 1,999, and *α*′ = *α*/*n*_gene_ = 2.5013 × 10^−5^. When applying the CMC method to scenario 1, we divide nonsynonymous SNPs in a gene into subgroups as follows: SNPs with MAFs in the interval (0, 0.01] form the first subgroup, and SNPs in the interval (0.01, 0.1) form the second subgroup; each remaining SNP with MAF ≥ 0.1 is treated as a subgroup by itself; all subgroups (variants) are analyzed using a Hotelling *T*^2^ test. When applying the CMC method to scenario 2, we divide nonsynonymous SNPs in a gene into three subgroups with SNPs having MAFs in the intervals (0, 0.001], (0.001, 0.01], and (0.01, 0.05].

Tables 2 and 3 show the results for scenarios 1 and 2, respectively. When all nonsynonymous SNPs are analyzed, the CMC method has the highest inflated FWER and weighted-sum method I has slightly inflated FWER compared to the nominal level of 0.05; the other two methods control the FWER well. On the other hand, when only nonsynonymous SNPs with MAF < 0.05 are analyzed, all four methods can control the FWER well.

**Table 2:** Results for the 200 data sets with all nonsynonymous SNPs

Association method	FWER	Power		
	
	*D*_4_	*D*_1_	*D*_2_	Disease
CMC	0.1150	0.1439	0.0062	0.0044
Weighed-sum method I	0.0550	0.1172	0.0065	0.0065
Weighted-sum method II	0.0400	0.1511	0.0042	0.0019
Variable threshold	0.0250	0.1306	0.0031	0.0064

**Table 3:** Results for the 200 data sets with nonsynonymous SNPs with MAF < 0.05

Association method	FWER	Power		
	
	*D*_4_	*D*_1_	*D*_2_	Disease
CMC	0.0100	0.1417	0.0023	0.0013
Weighted-sum method I	0.0150	0.1467	0.0015	0.0030
Weighted-sum method II	0.0100	0.1194	0.0023	0.0004
Variable threshold	0	0.1289	0.0031	0.0014

The FWER of the variable threshold method is 0 in Table 3. This is a little surprising. To verify the validity of the results, we also perform the standard permutation procedure with 10^6^ permutations. The standard permutation gives a FWER of 0.005 and exactly the same power as that of the fast permutation procedure. This shows again that the fast permutation method has slightly lower FWERs than the standard procedure does but has comparable average power. The reason that the FWER is 0 (or close to 0 in the standard permutation procedure) may be the small number of replicated data sets (200) and the conservative nature of the variable threshold. Tables 2 and 3 also show that all four methods have comparable power, which is low based on the analysis of the GAW17 data sets. No method has consistently better performance than other methods.

The CMC method is based on the Hotelling *T*^2^ test for subgroups of variants in a gene. Under the null hypothesis that no variants are associated with the disease, a key assumption for the Hotelling *T*^2^ test is that within each subgroup the proportion of individuals with at least one minor allele in case subjects is equal to the proportion in control subjects. When there is possible population stratification in the data as well as the existence of confounding variables such as Age or Smoking, the assumption may not be true and therefore the FWER of the Hotelling *T*^2^ test is inflated. The results also show that common variants are the main sources of the inflated FWERs (the CMC method does not have an inflated FWER when applied to SNPs with MAF < 0.05).

## Discussion

In this study, we evaluated four association tests for detecting rare variants by using the GAW17 data sets. When the rare variants and common variants were jointly analyzed, two methods (CMC and weighted-sum method I) had an inflated FWER; when only rare variants with MAF < 0.5 were analyzed, the four methods could control the FWER. It seems that including common variants in the analysis can increase the FWER of these four methods.

It is natural to ask why the average powers of these four methods are low for the analysis of the GAW17 data sets. There are three possible reasons. First, the phenotypes Q1 and Q2 do not completely depend on the genetic data. They also depend on other factors, such as age, sex, smoking history, and the correlation between Q1 and Q2. These factors may make it hard to detect the associated genes. Using a more general model that could account for these factors might improve the power. Second, the phenotypes are influenced by multiple genes. The effect of each gene may not be large, although the combined effects may be considerably significant. Testing a gene only individually certainly does not consider the aggregation effect of genes in the data. Third, population stratification and admixture may cause loss of power.

## Conclusions

For the simulated data sets of unrelated individuals in the GAW17 data, when the rare variants and common variants are analyzed jointly, the CMC method cannot control the FWER and weighted-sum method I has a slightly inflated FWER; when only the rare variants with MAF < 0.05 are analyzed, all methods control the FWER well. In both situations, the four methods have comparable power, which is low, because of the complexity of the GAW17 data. Using the fast permutation procedure for *p*-value estimation can produce FWERs and average powers close to those obtained using the standard permutation procedure.

## Competing interests

The authors declare that they have no competing interests.

## Authors’ contributions

WC and XG participated in the design of the study, did the simulation and drafted the manuscript. JW, CS, WW, DZ, NL, XC participated in the discussion of the study and revision of the manuscript. GG conceived of the study, and participated in designing the study, drafting and revising the manuscript. All authors read and approved the final manuscript.
